# The Prognostic Value of Baseline PSA Density on Long-term Oncological Outcomes in Men with an Initial Negative Prostate Biopsy: Results from ERSPC Rotterdam

**DOI:** 10.1016/j.euros.2026.06.006

**Published:** 2026-07-11

**Authors:** Meike J. van Harten, Sebastiaan Remmers, Ivo I. de Vos, Lionne D.F. Venderbos, Roderick C.N. van den Bergh, Monique J. Roobol, the ERSPC Rotterdam study group, F.H. Schroder, W.J. Kirkels, J.B.W Rietbergen, I.W. Koeter, R. Raaijmakers, S.H. de Vries, S. Roemeling, C. Gosselaar, T. Wolters, R.C.N. van den Bergh, P.J. van Leeuwen, M. Bul, X. Zhu, L.P. Bokhorst, A.R.Alberts, F-J. Drost, J.F.M. Verbeek, D.F. Osses, H.B. Luiting, R. Hogenhout, I.I. de Vos, R.C.A. Leenen, J.J. Lodder, M.J. van Harten, S.F. Westerhout, F. Denijs, E.F.H. Mulder, L.D.F. Venderbos, K. Beyer, H.A. van Vugt, G. Yurdakul, A. Boeken-Kruger, C. Wijburg, M. Forouzanfor, M. de Boer, R. Postma, A.N. Vis, Th van der Kwast, R. Hoedemaeker, G.J.L.H. van Leenders, R. van Schaik, P.J. van der Maas, H.J. De Koning, E.A. Heijnsdijk, S. Otto, G. Draisma, P. Beemsterboer, I. Korfage, R. Boer, M. Wildhagen, D. Nieboer, W. Merkelbach, W. Hoekstra, J. Blom, T. Lock, A. Noordzij, RAM Damhuis, A. Reedijk, R. Kranse, J. Helleman, D.W. Roobol, W. Roobol, E. van den Berg, G-J de Zwart, C.G.A.M. Franken-Raab, M. van Slooten-Midderigh, A. Smit, V van der Drift, E. de Bilde, Mden Rooijen, L. Mani, M. Visser-van Dongen, H. Versteeg-Leenheer, B. Zoutendijk, H. van Meurs, A.E. de Bruijn

**Affiliations:** Department of Urology, Erasmus MC Cancer Institute, University Medical Center Rotterdam, The Netherlands

**Keywords:** Prostate cancer, Screening, Negative biopsy, PSA density

## Abstract

**Introduction:**

Baseline prostate-specific antigen (PSA) is associated with the risk of metastatic disease (M+) and prostate cancer (PCa)-specific mortality (PCSM). However, the prognostic value of baseline PSA density (PSA-D) for long-term oncological outcomes is less well established. This study aims to evaluate the prognostic value of baseline PSA-D for oncological outcomes in men who participated in a population-based screening study and had an elevated PSA but a negative prostate biopsy at their first screening visit.

**Material and methods:**

Data from the Rotterdam cohort of the European Randomized Study of Screening for Prostate Cancer (ERSPC) were used. All men aged 55–69 yr with PSA ≥ 3 ng/ml following a negative (sextant) biopsy at initial screening were included. Screening was repeated every 4 yr, with (repeat) biopsy if PSA ≥ 3 ng/ml. Screen–detected PCa was defined as PCa diagnosed during scheduled screening rounds. Men were stratified into three age groups according to the maximum number of potential screening rounds: 55–59, 60–64, and 65–69, and three baseline PSA-D groups reflecting clinical practice: <0.10, 0.10–0.15, and ≥0.15 ng/ml^2^. Cumulative incidences (CIs) of indolent PCa, clinically significant PCa (csPCa) defined as Grade Group ≥2, M+ disease, and PCSM were assessed for each stratum at 21 yr after initial negative biopsy.

**Results:**

In total, 1565 men met the inclusion criteria: 55–59 (*n* = 368), 60–64 (*n* = 535), 65–69 (*n* = 662), with a median follow-up of 21 yr. A total of 268 men were diagnosed with PCa, of whom 66 had csPCa; 138 PCa cases were screen–detected, including 19 cases of csPCa. At 21 yr, the CI of M+ disease ranged from 0.3% to 5.2%, with the highest incidence in men aged 60–64 with PSA-D ≥ 0.15 ng/ml^2^ (5.2%, 95% CI: 1.9–11). PCSM was lowest in men with PSA-D < 0.10 ng/ml^2^, regardless of age group: <0.001% for men aged 55–59 and 65–69, 0.8% for men aged 60–64.

**Conclusions:**

Baseline PSA-D is a useful tool for identifying a group of men at low risk of future oncological events after an initial negative biopsy. Although continued screening may have contributed to low CI of oncological outcomes, the limited number of screen-detected csPCa during follow-up screening rounds supports the use of baseline PSA-D for risk stratification and de-escalation of repeat testing. Men aged 55–59 with a baseline PSA-D < 0.10 ng/ml^2^ after a negative prostate biopsy could be subject to extended screening intervals in a screening setting. Further validation in modern diagnostic settings is needed before firm clinical recommendations can be made.

## Take Home Message

Baseline prostate-specific antigen density is a useful tool in risk stratification aimed at identifying men at low risk for a future diagnosis of significant prostate cancer after a negative biopsy.

ADVANCING PRACTICE **What does this study add?**

The prognostic value of baseline prostate-specific antigen (PSA) is well established, but PSA density (PSA-D) has emerged as a stronger predictor of clinically significant prostate cancer, while its long-term prognostic role remains underexplored. This study demonstrates the long-term prognostic value of baseline PSA-D after a negative biopsy, reporting on 21 yr of follow-up. We found that baseline PSA-D is a useful tool in risk stratification aimed at identifying men at low risk for a future diagnosis of significant prostate cancer. Men with a baseline PSA-D < 0.10 ng/ml^2^ after a negative prostate biopsy could be subject to extended screening intervals or even excluded from further follow-up.

**Clinical Relevance**

This study is based on the Rotterdam ERSPC data and explores baseline PSA density, categorized into <0.10, 0.10–0.15, and ≥0.15, and its ability to predict long-term oncological outcome of prostate cancer such as development of metastasis and prostate cancer specific mortality. The study shows that baseline PSA density can be a powerful tool to stratify men for their long-term risk to develop advanced prostate cancer and, if the risk is high, the need of early detection measures and follow-up. Associate Editor: Jochen Walz.

**Patient Summary**

In this study, we looked at prostate cancer detection, progression, and mortality in men who had a negative prostate biopsy in the first round of a prostate cancer screening study. We had data with 21 yr of follow-up. By calculating and grouping PSA-D, a value calculated from a blood test (PSA) and the size of the prostate, we were able to identify a group of men who have a low risk of developing clinically significant prostate cancer (4.5%) or dying from prostate cancer (cumulative incidence <0.001 %). This means these men might be suitable for screening less often.

## 1 Introduction

Prostate-specific antigen density (PSA-D) has emerged as a statistically significant predictor for the detection of clinically significant prostate cancer (csPCa) [1], yet its prognostic value following a negative biopsy on long-term oncological outcomes remains underexplored. While a benign biopsy is an important negative predictive factor for harboring or developing csPCa, a proportion of men still might be at risk for clinically significant disease over time. Identifying which men might be at lower or higher risk for metastatic disease (M+ disease) or prostate cancer-specific mortality (PCSM) is crucial to decide post-biopsy follow-up intensity. This is particularly relevant in population-based screening programs, where efficient risk stratification is essential to strike a balance between early detection and limited resources, but could certainly also be applied in clinical situation.

Multiple studies have addressed baseline prostate-specific antigen (PSA), defined as the initial PSA measurement, and its impact on long-term oncological outcomes [2], [3], [4], [5], [6], [7]. According to current European Association of Urology (EAU) guidelines, PSA testing should be repeated after a negative screening [8]. However, the optimal interval for these PSA tests remains uncertain, with intervals ranging between 2 and 8 yr in a screening setting. In addition, men with a negative biopsy may have a different risk profile than those with a negative magnetic resonance imaging (MRI) result or elevated PSA, suggesting that distinct follow–up intervals may be warranted. This highlights the need for further research into baseline characteristics after a negative biopsy to guide follow–up.

This study aims to retrospectively determine the prognostic value of baseline PSA-D for oncological outcomes across different age groups of men with a baseline PSA ≥ 3 ng/ml and a negative prostate biopsy, using long-term follow-up of the European Randomized Study of Screening for Prostate Cancer (ERSPC), Rotterdam section.

## 2 Patients and methods

A retrospective cohort study was performed using data from the Dutch (Rotterdam) arm of the ERSPC, which has been described in detail previously [9], [10]. In short, men aged 55–74 yr were randomized into either a screening arm or a control arm based on a 1:1 trial arm allocation following the provision of informed consent. Participants in the screening arm underwent repeated PSA testing every 4 yr, with the intent to participate up to the age of 74 yr if no prostate cancer (PCa) detection occurred, digital rectal examination, and transrectal ultrasound (TRUS). Except for the initial half of the first screening round, a PSA threshold of ≥3 ng/ml was applied as an indication for lateralized systematic TRUS-guided sextant prostate biopsies. Cases of PCa detected and deaths were available through regular linkages with the Netherlands Cancer Registry and Statistics Netherlands. PCSM was evaluated by a cause-of-death committee and defined as death that was: (1) definitely PCa-related, (2) intervention-related, or (3) probably PCa-related [11].

In this study, we included men from the predefined ERSPC core-age group (55–69 yr) who had a PSA ≥ 3 ng/ml, followed by a negative sextant systematic lateralized prostate needle biopsy at their first screening.

### 2.1 Statistical analyses

For the analyses, the study cohort was divided into three different age groups: 55–59, 60–64, and 65–69 yr at the time of the first screening round. These age categories reflect the maximum number of screening rounds each group could have undergone, considering the upper age limit of screening (74 yr) and a 4-yr screening interval, resulting in a maximum of five, four, and three screening rounds, respectively. The TRUS prostate volume (ml) combined with PSA level (ng/ml) at initial screening was used for calculating PSA-D. TRUS was performed by specifically trained staff, and volumes were measured using 5-mm step-section planimetry. A subdivision was made within each age group for baseline PSA-D: ≤0.10, 0.10–0.15, and ≥0.15 ng/ml^2^, based on cutoffs frequently applied in EAU guidelines [8], with ≥0.15 ng/ml^2^ commonly applied as a biopsy trigger, <0.10 ng/ml^2^ considered low–risk, and 0.10–0.15 ng/ml^2^ representing a clinically relevant so-called “gray-zone”.

Within each age and PSA-D group, the following outcomes were calculated up to 21 yr after initial screening: cumulative incidence of indolent PCa (indPCa) and csPCa (censoring men diagnosed with indolent PCa), metastatic disease (both metastases at diagnosis and progression to distant metastases during follow-up) (all using any death as a competing risk), and PCSM (using other-cause mortality as a competing risk) [12]. Cancer detection encompassed PCa cases identified during screening (screen-detected), between screening rounds (<4 yr after the previous screening round, so-called interval cancers), in nonattenders (men who did not participate in subsequent screening rounds), or after the protocolized screening program, which ended at age 74. Men who were alive at the fixed end date (January 1, 2022) were censored, as were events (PCa, M+, and death) that occurred after 21 yr of follow-up, whichever came first. For csPCa, defined as Gleason ≥ 7 (graded according to the pre-2005 International Society of Urological Pathology criteria, corresponding to ISUP ≥2), censoring also included men who were diagnosed with ISUP Grade Group (GG) 1 PCa.

Missing GG scores were addressed using two separate methodological approaches, tailored to the type of analysis. For descriptive statistics, missing GG scores were complemented based on the EAU classification, in which indolent PCa is defined as stage <cT2b and PSA < 10 ng/ml [8]. The remaining cases were classified as csPCa [8]. For the cumulative incidence analyses, missing data were handled using multiple imputation by chained equations (MICE). The imputation of missing GG scores was performed based on cT stage and PSA levels. Ten imputed datasets were generated and subsequently pooled for analysis following Rubin’s rules.

To assess the potential impact of baseline PSA-D on follow-up screening, we evaluated outcomes associated with refraining from subsequent PSA testing in men with PSA-D < 0.10 ng/ml^2^. This analysis was stratified by age group to explore differential effects across age categories.

To evaluate whether the effect of PSA-D on PCSM was similar for each age group and to quantify the effect of PSA-D continuously, we used a Cox proportional hazards model to evaluate the continuous effect of PSA-D and age group on PCSM. We evaluated whether adding an interaction term between PSA-D and age group and a nonlinear relationship of PSA-D improved the model fit using the likelihood ratio test.

All statistical analyses were performed using R (v4.3.2, R Foundation for Statistical Computing, Vienna, Austria).

## 3 Results

A total of 1565 men met the inclusion criteria. Among these men, 368 (24%) were aged 55–59 yr, 535 (34%) were aged 60–64 yr, and 662 (42%) were aged 65–69 yr (Table 1). The median follow-up of those still at risk was 21 yr (interquartile range: 21–21), with 790 men still at risk at the end of follow-up. GG of PCa detected after the first screening round was missing for 37 out of 268 men diagnosed with PCa (14%).

**Table 1:** Cumulative incidence of indolent prostate cancer (indPCa), clinically significant prostate cancer (csPCa) detection, metastatic disease (M+), and prostate cancer-specific mortality (PCSM) at 21 yr follow-up for different age groups sub-categorized in different baseline prostate-specific antigen density groups

Age- and PSA-D group	Men (*N*)	*N* indPCa	Cumulative incidence indPCa (%)	*N* csPCa	Cumulative incidence csPCa (%)	*N* M+	Cumulative incidence M+ (%)	*N* PCSM	Cumulative incidence PCSM (%)
Total	1565	202		66		24		14	
55–59									
<0.10	143	23	17 (11–24)	3	2.5 (0.7–6.6)	2	0.7 (0.06–3.5)	0	0.0 (0.0–0.0)
0.10–0.15	134	28	23 (16–31)	8	6.8 (3.2–12)	3	1.5 (0.3–4.8)	0	0.0 (0.0–0.0)
≥0.15	91	19	22 (13–32)	6	8.7 (3.5–17)	3	3.3 (0.9–8.6)	2	2.2 (0.4–7.0)
60–64									
<0.10	258	31	13 (9.4–18)	7	3.3 (1.4–6.5)	1	0.4 (0.04–2.0)	3	0.8 (0.2–2.6)
0.10–0.15	180	22	13 (8.0–18)	10	6.9 (3.5–12)	2	1.1 (0.2–3.7)	3	1.7 (0.5–4.5)
≥0.15	97	15	17 (9.6–25)	11	13 (6.4–22)	5	5.2 (1.9–11)	3	2.1 (0.4–6.6)
65–69									
<0.10	334	25	8.2 (5.3–12)	9	3.8 (1.8–7.1)	2	0.3 (0.03–1.6)	0	0.0 (0.0–0.0)
0.10–0.15	202	23	9.4 (5.5–15)	9	5.9 (2.4–12)	3	0.5 (0.05–2.6)	2	0.5 (0.05–2.6)
≥0.15	126	16	12 (6.2–19)	3	4.3 (0.7–14)	3	2.4 (0.6–6.3)	1	0.8 (0.07–4.0)

PSA-D = PSA-density; indPCa = indolent prostate cancer; csPCa = clinically significant prostate cancer; M+ = metastatic disease; PCSM = prostate cancer-specific mortality

### 3.1 Prostate cancer incidence

At 21 yr of follow–up after the initial negative prostate biopsy, 268 men were diagnosed with PCa. Of these, 138 cases were detected through organized screening (screen-detected), including 19 clinically significant cancers (SD-csPCa). In total, 66 of the 268 cancers were classified as csPCa and 202 as indolent. Supplementary I, II, and III display, for each age group and baseline PSA-D group, the number of cancers detected by the number of performed screening rounds and those that emerged after nonattendance or during the screening interval. In addition, the number of PCa cases that were detected and consequently progressed to M+ disease and led to PCSM are shown per screening round.

At 21 yr of follow-up, the highest cumulative incidence for indPCa was observed in men aged 55–59 yr with a baseline PSA-D of 0.10–0.15 ng/ml^2^ (23%, 95% confidence interval [CI]: 16–31); for csPCa, the highest cumulative incidence was found for men aged 60–64 with a PSA-D ≥ 0.15 ng/ml^2^ (13%, 95% CI: 6.4–22) (Table 1). Across all age groups, men with a PSA-D < 0.10 ng/ml^2^ were consistently associated with the lowest cumulative probabilities on both indPCa and csPCa.

### 3.2 Continuous PSA-D and age group on PCSM

The Cox proportional hazards model using the main effects of PSA-D and age-group did not statistically improve when an interaction was added (*p* = 0.5). In addition, restricted cubic splines (3 knots showed the lowest Akaike information criterion), did not statistically improve the model either (*p* = 0.2). After correction for PSA-D, there were no statistically significant differences in PCSM between men aged 60–64 versus 55–59 yr (hazard ratio [HR] = 3.11; 95% CI: 0.63–15.3; *p* = 0.2) and men aged 65–69 versus 55–59 yr (HR = 0.99; 95% CI: 0.14–7.29, *p* > 0.9). The continuous effect of PSA-D on PCSM at 21 yr for each age group is presented in Supplementary IV.

### 3.3 Metastatic disease and PCSM

Of 268 men diagnosed with PCa, 24 cases progressed to M+ disease. Across all age groups, the highest cumulative incidence for M+ disease was observed in men with PSA-D ≥ 0.15 ng/ml^2^, with probabilities of 3.3% (95% CI: 0.9–8.6) for men aged 55–59 yr, 5.2% (95% CI: 1.9–11) for men aged 60–64 yr and 2.4% (95% CI: 0.6–6.3) for men aged 65–69 yr. However, it must be noted that the number of cases of M+ disease within each age and PSA-D subgroup was relatively small.

PCSM was consistently lowest in men with low baseline PSA-D (<0.10 ng/ml^2^), regardless of age group. Within each age category, the probability of PCSM increased with higher baseline PSA-D values. A total of 14 men died of PCa. Among men aged 55–59 yr with PSA-D <0.10 and 0.10–0.15 ng/ml^2^, and men aged 65–69 yr with PSA-D <0.10 ng/ml^2^, no men died of PCa.

### 3.4 Effect of baseline PSA-D on follow-up screening efficiency

Table 2 displays the various age group combinations with baseline PSA-D < 0.10 ng/ml^2^, highlighting the potential for reduced follow-up diagnostics and their associated outcomes.

**Table 2:** Display of the impact of different age-groups with PSA-D < 0.10 ng/ml^2^ on potential screening reduction and corresponding oncological consequences

	Men, *N*	PSA measurements, *N*	Biopsies, *N*	csPCa cases
Total	1565	1714	1269	66
Potential reduction				
Age- and baseline PSA-D threshold	Potential reduction in men requiring follow-up, *N*	Potential reduction in PSA measurements during FU, *N*	Potential reduction in biopsies during follow-up, *N*	Missed csPCa cases, *N*
Age 55–59, PSA-D < 0.10 ng/ml^2^	143	210	166	3
Age 55–59, 60–64, PSA-D < 0.10 ng/ml^2^	401	577	433	10
Age 55–59, 60–64, 65–69 PSA-D < 0.10 ng/ml^2^	735	816	625	19

PSA-D = PSA-density; csPCa = clinically significant prostate cancer; FU = follow-up.

Over a 21-yr follow-up after the initial negative biopsy, deintensifying follow-up in men aged 55–59 yr with PSA-D < 0.10 ng/ml^2^ has the potential to reduce further PSA testing by 143 men (9.1% from total cohort [*N* = 1565]), resulting in 210 fewer PSA screenings and 166 fewer biopsies, while missing 3 cases of csPCa (4.5% from the total number of csPCa cases [*N* = 66]).

Extending this group to include men aged 60–64 yr with PSA-D < 0.10 ng/ml^2^ could increase the number of men with deintensified follow-up testing to 401 (26%), with 577 fewer PSA measurements and 433 fewer biopsies, missing 10 csPCa cases (15% of total screen-detected csPCa). Considering the entire age range in this study (55–69 yr) with PSA-D < 0.10 ng/ml^2^ could deintensify follow-up testing in 735 men (47%), with 816 fewer PSA measurements and 625 fewer biopsies, at the cost of missing 19 csPCa cases (29% of total csPCa) over 21 yr.

## 4 Discussion

We assessed whether baseline PSA-D can stratify men for oncological events following a first screening round with elevated PSA ≥ 3 ng/ml and a negative initial biopsy. First, it must be noted that the overall incidence of M+ disease and PCSM was low in this study cohort. Among the 1565 included men, 24 developed M+ disease and 14 died from PCa. These findings align with previous studies assessing long-term oncological outcomes after a negative biopsy [13], [14], [15]. This is further supported by a systematic review by Kawa et al, which summarized PCSM after negative biopsies in screening studies and reported similarly low PCSM cumulative incidences (1.8–5.2%) with follow-up ranging from 10 to 20 yr, regardless of study protocol differences [16]. Collectively, these studies highlight that the optimal follow-up after a negative biopsy is still uncertain.

Our study has shown that age does not affect PSA-D and that PSA-D can be used to identify a subgroup within the entire cohort that has an even lower probability of oncological events. The results per PSA-D category showed that men with a PSA-D < 0.10 ng/ml^2^ have the lowest cumulative incidence for csPCa, M+ disease, and PCSM compared with the other PSA-D categories within the same age group. Specifically, men aged 55–59 yr with baseline PSA–D < 0.10 ng/ml^2^ after a negative biopsy could be considered for longer follow-up intervals, which in this cohort has the potential to reduce additional testing by 9.1% at the cost of missing 4.5% of all csPCa (3 cases; 0 PCSM). This is particularly important in a population-based screening context, where efficient allocation of clinical resources is essential.

Pylvalainen et al also investigated the association between PSA-D and PCSM after a benign systematic prostate biopsy in the Finnish cohort of the ERSPC [17]. Similar to our results, they found that the overall probability of PCSM after a negative biopsy is low at 20 yr follow-up (2.1%). However, their estimate was higher compared with our cohort (0.9%) and is likely explained by variation in screening intensity, among other factors: men in the Finnish arm underwent fewer screening rounds on average (mean, 1.6) than those in the Dutch arm (mean, 2.3) [18]. More frequent screening increases the chance of earlier detection and, as a result, a higher probability of cure. Notably, in their study, the probability of PCSM was lower in men with PSA-D < 0.15 ng/ml^2^ compared with men with PSA-D ≥ 0.15 ng/ml^2^ (0.5%, 95% CI: 0.2–1.1 vs 2.0%, 95% CI: 1.2–3.1). This aligns with our results, which showed that the probability of PCSM increased with higher baseline PSA-D across all age groups.

Our findings show that PSA-D provides prognostic information for follow-up intensity in men with an elevated PSA but a negative biopsy. Defining a rescreening interval is not only important to avoid unnecessary testing but also crucial in reaching the effect we want to achieve with screening, that is, a relevant reduction in PCSM. Participants in our cohort underwent repeated screening rounds, which could have facilitated earlier detection and treatment of csPCa, thereby reducing the incidence of late-stage disease and mortality. Nonetheless, the number of screen-detected csPCa cases in follow-up rounds remained relatively low (*N* = 19), suggesting that the risk of missed aggressive cancers at baseline was limited. This supports the use of baseline PSA-D as a valuable tool for risk stratification and for informing appropriate screening intervals.

To the best of our knowledge, this is the first study to examine long-term oncological outcomes (both M+ disease and PCSM) and the prognostic value of PSA-D in men with a first negative prostate biopsy within specific age groups, based on data from a large, randomized controlled trial with high-quality follow-up data. However, several limitations should be mentioned. First, in this cohort, a well-known flaw in studies with long follow-up is that the applied diagnostic algorithm is different from current guidelines. MRI was not utilized for risk stratification between PSA measurement and biopsy, which may have led to missed MRI-visible csPCa that was not detected by sextant biopsy alone. However, a previous study from our study group, evaluating the same cohort after 11 yr, found that lateralized sextant biopsies are not obsolete when repeated screening is applied [19]. Moreover, the low overall PCa detection rate observed in this cohort after 21 yr makes it unlikely that the biopsy method affected these findings. Complementary to this, the Goteborg-2 population-based screening trial showed that omitting biopsies in men with negative MRI reduces the detection of indolent PCa by 50% while maintaining a comparable csPCa detection rate [20]. This demonstrates that MRI–integrated pathways reduce unnecessary biopsies and limit the detection of indolent PCa, resulting in a more selective post–MRI diagnostic profile and, consequently, a higher-risk biopsy cohort. Within this contemporary diagnostic context, our findings can be interpreted to indicate that PSA–D retains important discriminatory value even after MRI triage. For example, men with PSA–D > 0.20 ng/ml^2^ and a negative biopsy, those with Prostate Imaging Reporting and Data System (PI-RADS) 1–2 have a lower residual risk than those with PI-RADS 4–5.

Second, the number of oncological events observed in this study was limited. This confirms that the overall probability of adverse oncological outcomes after a negative biopsy is low, yet it also reduces the precision of the estimates. Furthermore, information on GG was missing for 14% of the detected cases. Missing data were imputed as described in the Results. Since the overall PCa detection was low, the absolute number of cases with missing GG information was small. Therefore, the impact of imputing these values on the overall findings is expected to be limited.

Third, due to differences in potential screening rounds across age groups, no direct comparisons can be made in terms of the risk of M+ disease and PCSM. For this reason, we restricted our analyses to comparisons within each age group across the different PSA–D categories. Moreover, using age–specific cutoffs is appropriate, as men aged 50 yr at their first screening have different probabilities of oncological outcomes compared with men aged 70 yr at their first screening.

## 5 Conclusion

To conclude, these long-term outcomes show the association between baseline PSA-D with significant and indolent PCa, M+ disease, and PCSM across different age groups in a screening trial. PSA-D after an elevated PSA but a negative biopsy can effectively identify a cohort (men aged 55–59 with PSA-D < 0.10 ng/ml^2^) that could be considered for extended screening intervals. This approach would lead to only high-risk men after a negative biopsy continuing with frequent follow-up visits. Consequently, this conserves health care resources by reducing the number of screening visits and further diagnostic follow-up, while maintaining a minimal risk of missing M+ disease and PCSM in the excluded group. Further validation in modern diagnostic settings is needed before firm clinical recommendations can be made.

  ***Author contributions***: Meike J. van Harten had full access to all the data in the study and takes responsibility for the integrity of the data and the accuracy of the data analysis.

  *Study concept and design:* van Harten, Remmers, de Vos, van den Bergh, Roobol.

*Acquisition of data:* Supplementary.

*Analysis and interpretation of data:* van Harten, Remmers.

*Drafting of the manuscript:* van Harten, Remmers.

*Critical revision of the manuscript for important intellectual content:* van Harten, Remmers, de Vos, Venderbos, van den Bergh, Roobol.

*Statistical analysis:* van Harten, Remmers.

*Obtaining funding:* None.

*Administrative, technical, or material support:* None.

*Supervision:* Roobol.

*Other:* None.

  ***Financial disclosure:*** Meike J. van certifies that all conflicts of interest, including specific financial interests and relationships and affiliations relevant to the subject matter or materials discussed in the manuscript (eg, employment/affiliation, grants or funding, consultancies, honoraria, stock ownership or options, expert testimony, royalties, or patents filed, received, or pending), are the following: None.

  ***Funding/Support and role of the sponsor:*** None.

## Appendix A Supplementary material

Supplementary Data 1 Supplementary I, II, II - Flowcharts showing oncological outcomes across screening rounds following an initial negative biopsy, stratified by age- and PSA density group. Supplementary IV - Association between PSA Density and Prostate cancer specific mortality across age groups.
