# Supplementary material for: The Prognostic Value of Baseline PSA Density on Long-term Oncological Outcomes in Men with an Initial Negative Prostate Biopsy: Results from ERSPC Rotterdam
Source: Eur Urol Open Sci. 2026 Jul 11;90:131–6. doi: 10.1016/j.euros.2026.06.006 (PMC13382057; doi:10.1016/j.euros.2026.06.006)
Supplement: Supplementary Data 1 — Supplementary I, II, II - Flowcharts showing oncological outcomes across screening rounds following an initial negative biopsy, stratified by age- and PSA density group. Supplementary IV - Association between PSA Density and Prostate cancer specific mortality across age groups. [file mmc1.docx]

**Supplementary I**

*
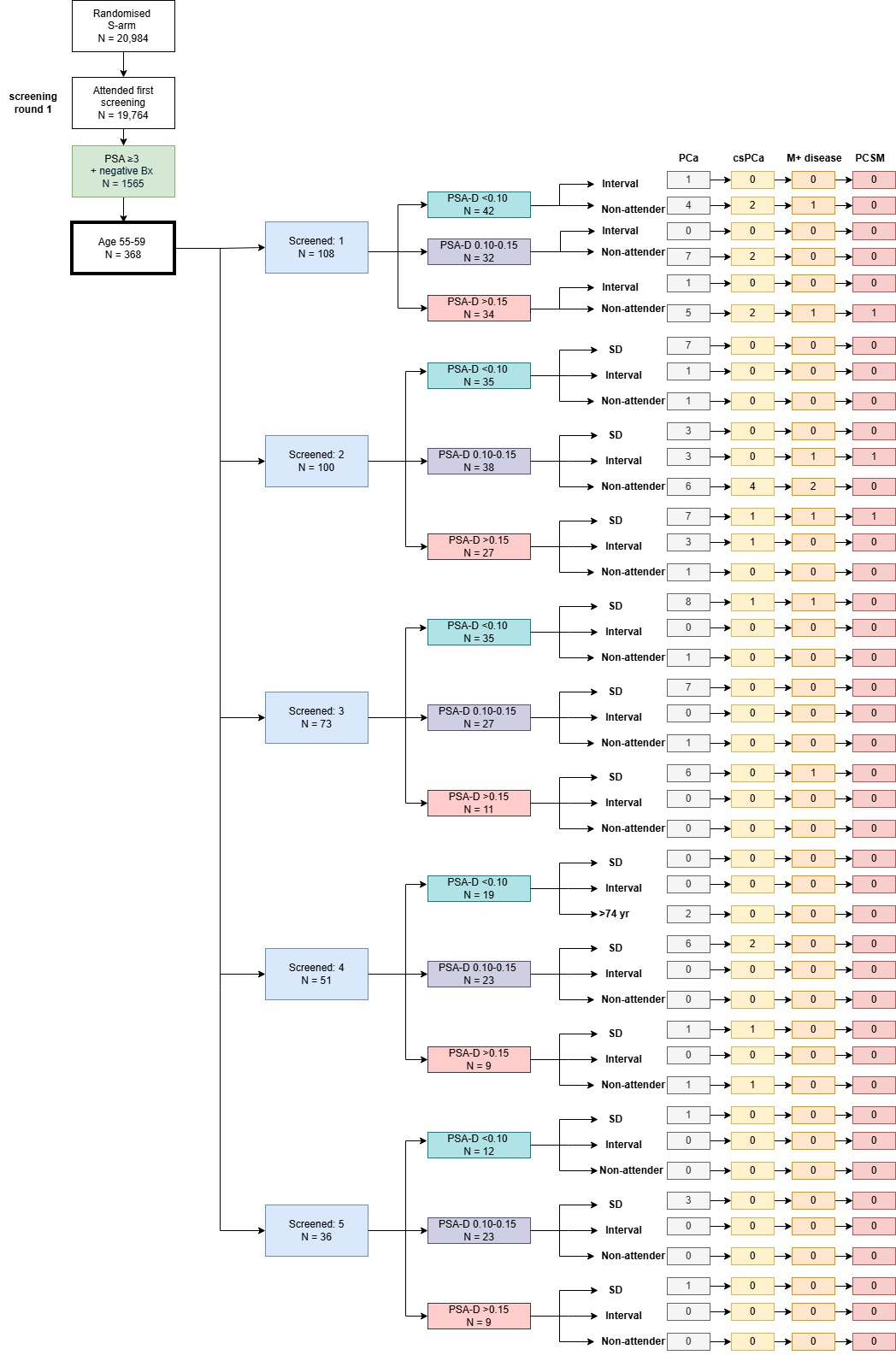
* Supplementary figure 1. Flowchart for men aged 55-59 at first negative biopsy, showing the detection of PCa, progression to M+ disease and PCSM for number of performed screening-rounds within each PSA-density group. S-arm = screening-arm, PCa = prostate cancer, csPCa = clinically significant prostate cancer, M+ disease = metastastic disease, PCSM = prostate cancer specific mortality, PSA-D = psa-density, SD = screen-detected

**Supplementary II**


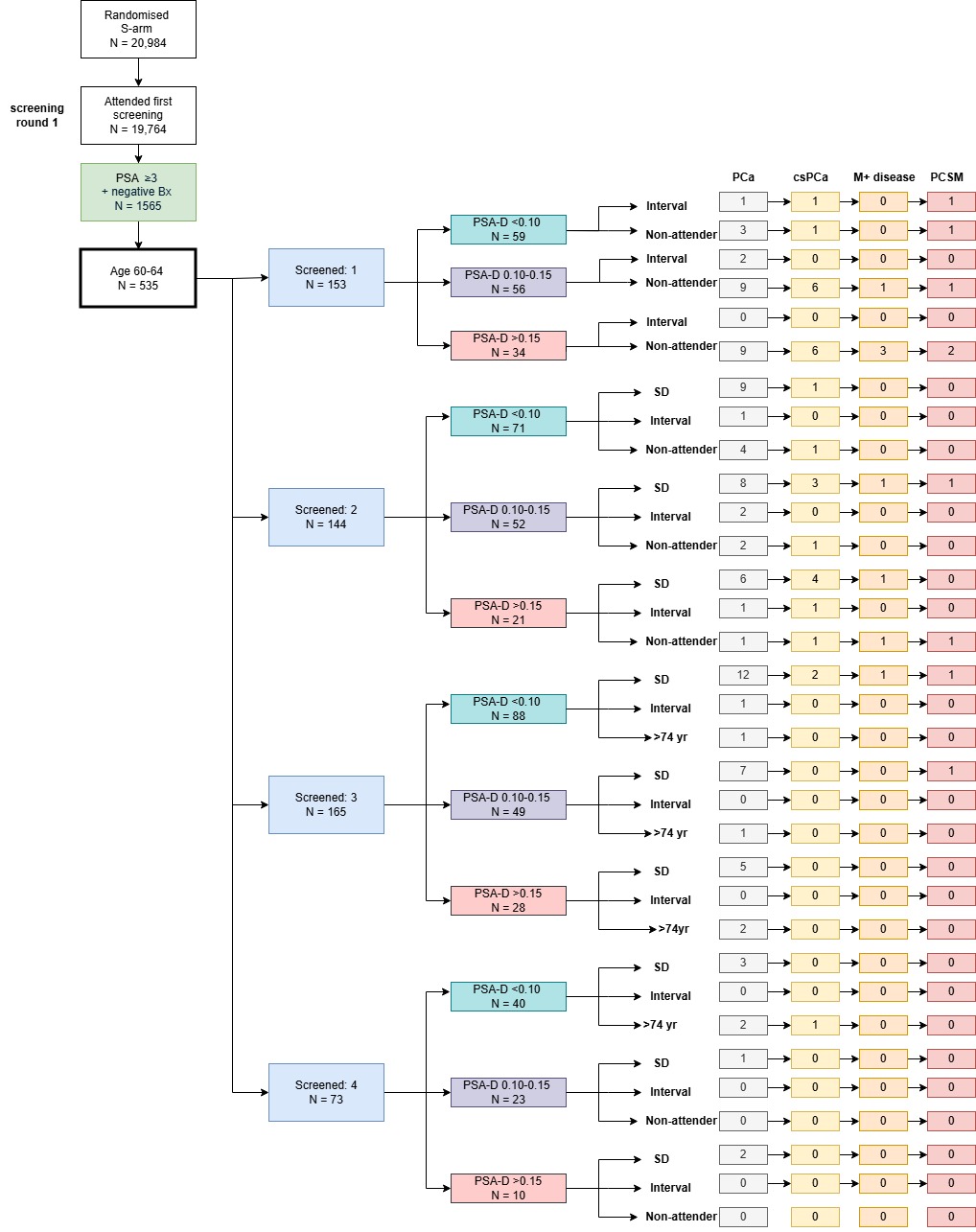


Supplementary figure 2. Flowchart for men aged 60-64 at first negative biopsy, showing the detection of PCa, progression to M+ disease and PCSM for number of performed screening-rounds within each PSA-density group.

S-arm = screening-arm, PCa = prostate cancer, csPCa = clinically significant prostate cancer, M+ disease = metastastic disease, PCSM = prostate cancer specific mortality, PSA-D = psa-density, SD = screen-detected

**Supplementary III**


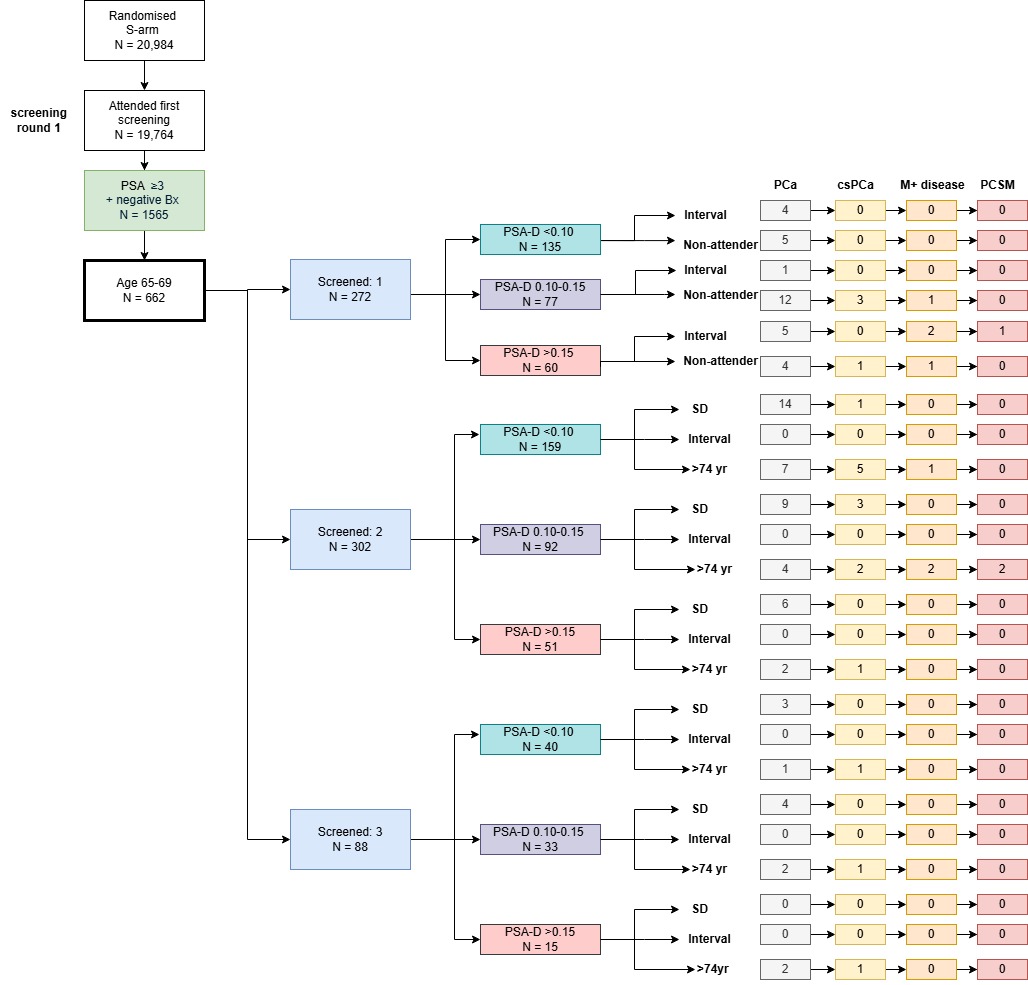


Supplementary figure 3. Flowchart for men aged 65-69 at first negative biopsy, showing the detection of PCa, progression to M+ disease and PCSM for number of performed screening-rounds within each PSA-density group.

S-arm = screening-arm, PCa = prostate cancer, csPCa = clinically significant prostate cancer, M+ disease = metastastic disease, PCSM = prostate cancer specific mortality, PSA-D = psa-density, SD = screen-detected

**Supplementary IV**


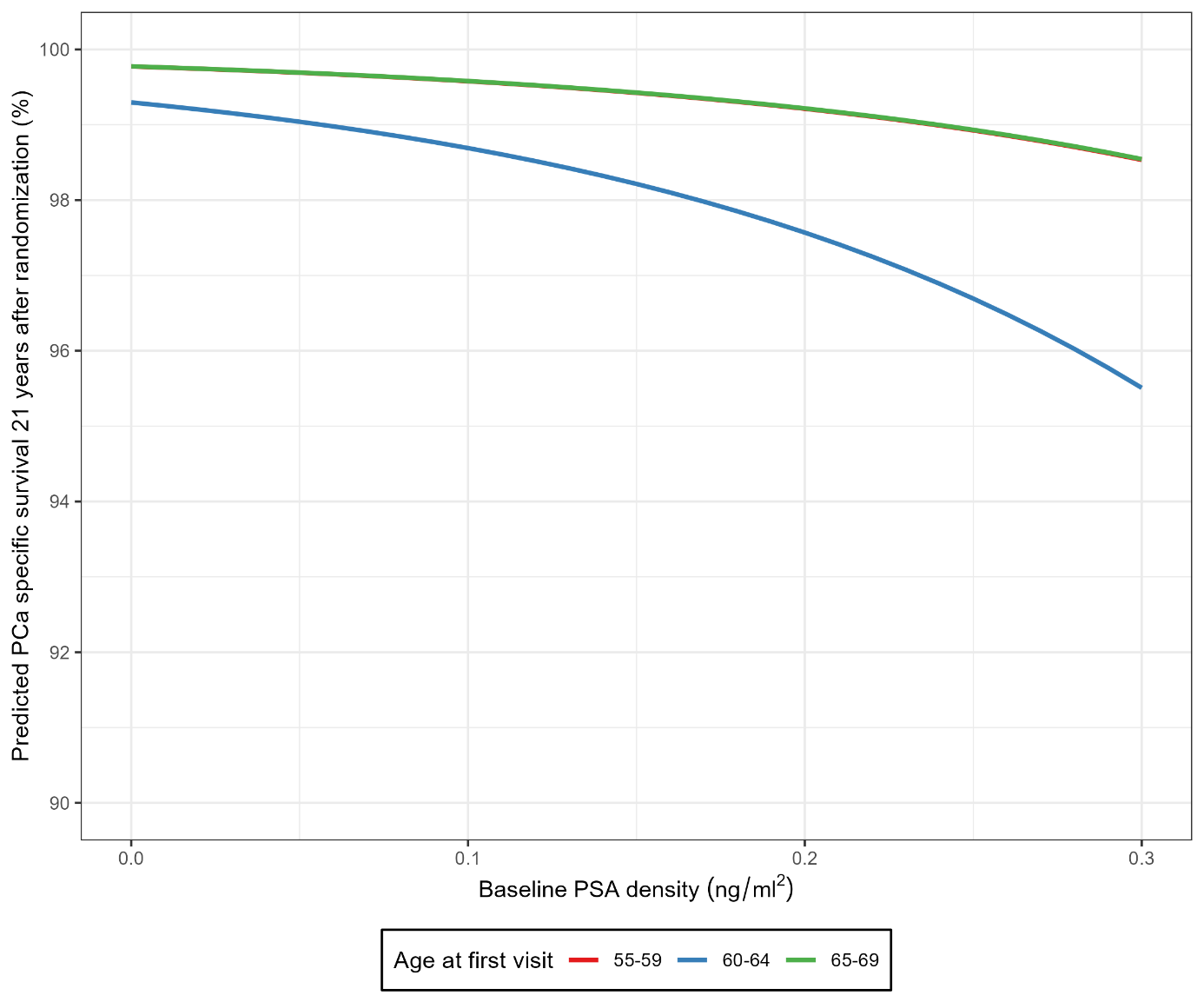


**Supplementary V : ERSPC investigators**

The following people are being acknowledged for their contribution to the ERSPC study.

**The Netherlands:**

Prof. Dr. F.H. Schroder

Dr. W.J. Kirkels, urologist

Dr. J.B.W Rietbergen, urologist

Dr. I.W. Koeter, urologist

Dr. R. Raaijmakers, urologist

Dr. S.H. de Vries, urologist

Dr. S. Roemeling, urologist

Dr. C. Gosselaar, general practicioner

Dr. T. Wolters, urologist

Dr. R.C.N. van den Bergh, urologist

Dr. P.J. van Leeuwen, urologist

Dr. M. Bul, PhD urologist

Dr. X. Zhu, PhD urologist

Dr. L.P. Bokhorst, radiologist

Dr. A.R.Alberts, urologist

Dr. F-J. Drost, GP

Dr. J.F.M. Verbeek, Radiologist

Dr. D.F. Osses, Urologist

Dr. H.B. Luiting, Urologist

Dr. R. Hogenhout, Urologist

Drs. I. I. de Vos, Urologist

Drs. R.C.A. Leenen, PhD student

Drs. J. J. Lodder, PhD student

Drs. M.J. van Harten, PhD student

Drs. S.F. Westerhout, PhD student

Drs. F. Denijs, PhD student

E.F.H. Mulder, MSc student

Dr. L.D.F. Venderbos, quality of life researcher

Dr. K. Beyer, postdoctoral researcher

Dr. H.A. van Vugt

Drs. G. Yurdakul, urologist

Drs. A. Boeken-Kruger, urologist

Drs. C. Wijburg, urologist

Drs. M. Forouzanfor, urologist

Drs. M. de Boer, urologist

Dr. R. Postma, pathologist

Prof. Dr. A.N. Vis, urologist

Prof. Dr. Th van der Kwast, pathologist

Dr. R. Hoedemaeker, pathologist

Prof. Dr. G.J.L.H. van Leenders, pathologist

Prof. Dr. R. van Schaik, clinical chemistry

Prof. Dr. P.J. van der Maas, epidemiologist

Prof. Dr. H.J. De Koning, epidemiologist

Dr. E.A. Heijnsdijk, epidemiologist

Dr. S. Otto, epidemiologist

Dr. G. Draisma, statistician

Dr. P. Beemsterboer, epidemiologist

Dr. I. Korfage, epidemiologist

Dr. R. Boer, informatician/mathematician

Dr. M. Wildhagen, statistician

Drs. D. Nieboer, statistician

Drs. W. Merkelbach, COD committee

Dr. W. Hoekstra, COD committee

Dr. J. Blom, COD committee

Dr. T. Lock, COD committee

Dr. A. Noordzij, COC committee

Drs. RAM Damhuis, epidemiologist, cancer registry

Drs. A. Reedijk, epidemiologist, cancer registry

Dr. R. Kranse, statistician, cancer registry

Dr. J. Helleman, data management

Mrs. D.W. Roobol, data management

Mr. W. Roobol, website management

Mrs. E. van den Berg, administrative support

Mr. G-J de Zwart, echografist

Mrs. C.G.A.M. Franken-Raab, data management

Mrs. M. van Slooten-Midderigh, data management

Mrs. A. Smit, medical assistant

Mrs. V van der Drift, data management

Mrs. E. de Bilde, data management

Mrs. M den Rooijen, data management

Mrs. L. Mani, medical assistant

Mrs. M. Visser-van Dongen, data management

Mrs. H. Versteeg-Leenheer, data management

Mrs. B. Zoutendijk, data management

Mrs. H. van Meurs, echografist

Mrs. A E. de Bruijn, secretary
